# Moral sensitivity and its dimensions in Iranian nursing students

**Published:** 2016-12-27

**Authors:** Fariba Borhani, Abbas Abbaszadeh, Mohammad Javad Hoseinabadi-Farahani

**Affiliations:** 1 Associate Professor, Medical Ethics and Law Research Center, Shahid Beheshti University of Medical Sciences, Tehran, Iran;; 2 Professor, Department of Medical Surgical Nursing, Shahid Beheshti University of Medical Sciences, School of Nursing and Midwifery, Tehran, Iran; Iranian Academy of Medical Sciences, Tehran, Iran;; 3 PhD Student, Department of Nursing, University of Social Welfare and Rehabilitation Sciences, Tehran, Iran.

**Keywords:** *Ethics*, *Moral sensitivity*, *Nursing*, *Nursing students*

## Abstract

As future providers of health services, nursing students should learn about ethical concepts over the course of their education. These concepts are currently taught in nursing schools, yet the degree of moral sensitivity in nursing students before entering clinical settings is a topic of controversy. This was a cross-sectional study on the nursing students studying for a bachelor’s degree in Qazvin University of Medical Sciences selected through census sampling (n = 205). Data were collected by Lutzen's Moral Sensitivity Questionnaire and analyzed through statistical tests using SPSS 16. The level of significance was *P *< 0.05. In order to conduct the study, permission was obtained from the Ethics Committee of Shahid Beheshti University of Medical Sciences.

The mean of moral sensitivity was found to be 66.1 + 8.1, which is a moderate level. Of all the dimensions of moral sensitivity, "expressing benevolence" had the highest (16.9 ± 4.04) and "structuring moral sense" had the lowest (5.2 ± 1.45) mean scores. Among demographic variables, age was found to have a significant positive correlation with the score of moral sensitivity (r = 0.2, *P* = 0.01).

Nursing students are relatively familiar with the ethical concepts of patient care, but that does not seem to be sufficed, as moral sensitivity is an extremely crucial factor in care. It is therefore recommended that the necessary training be provided to develop moral sensitivity in nursing students both in educational and practical environments.

## Introduction

Healthcare systems across the world have experienced extensive changes in recent years. Some instances include technological advances, the discovery and incidence of emerging diseases, new diagnostic techniques and tools, developments in care processes and medical interventions, population growth, increased rate of migration, and shortage of nurses. In line with these changes, awareness of patients’ rights has also increased within healthcare organizations (1-3). As an integral part of healthcare systems, the nursing profession focuses on care and the promotion of community health (4).

Understanding ethics as a profound and complex process lived by people is a basic nursing action. At first glance, nursing practices and competencies may appear as clinical procedures occurring independently of ethics; however, ethics and clinical procedures are practically inseparable (5-7). 

As future health care providers, nursing students should learn about ethical concepts related to nursing practice over the course of their education. The purpose of teaching ethics to students is to foster nurses who are able to apply ethics to their care and to develop and promote ethical decision-making skills in the face of ethical dilemmas (7-9).

According to Lutzen et al., ethical decision-making comprises four components: 1) moral sensitivity; 2) moral judgment; 3) moral motivation; and 4) moral character (10). Of these four components, moral sensitivity is the most important, since it enables nurses to identify ethical problems in providing patient care, to make the most ethical decision and to achieve ethical sensitivity (11-14).

Like other developing countries, nurses in Iran are faced with various challenges, including job dissatisfaction, poor social standing, shortage of nurses, and the gap between theory and practice. Each of the above-mentioned problems can affect the quality of nursing care (6) and lead to wrong decisions resulting from work pressure. Ethical decision-making is not only contingent upon nurses’ proper management of clinical ethical issues (15-17), but also necessitates moral sensitivity (18, 19). In fact, the latter is a prerequisite for ethical decision-making (20).

Moral sensitivity and reasoning are considered crucial nursing skills required for ethical decision-making and problem-solving and their dimensions in clinical settings (19, 21). For an effective application of ethics, nurses should develop their powers of ethical reasoning, understanding and analysis as well as their moral sensitivity (22).

Moral sensitivity is an attribute that enables the individual to recognize ethical conflicts, to have a sensory and intellectual understanding of people in vulnerable situations, and to develop an awareness of the ethical consequences of making decisions about others. Moral sensitivity requires care providers to recognize and interpret the spoken and non-spoken behaviors and signs expressed by patients so as to recognize their needs (23-27). As future providers of health services, nursing students should learn about ethical concepts over the course of their education. These concepts are currently taught in nursing schools; however, the controversial issue is the degree to which nursing students possess moral sensitivity before entering clinical settings (28, 29).

Studies conducted in Iran as well as other countries on moral sensitivity in nurses have indicated different levels of this moral concept (30-32). Ahn and Yeom reported poor levels of moral sensitivity among Korean nursing students and argued that teaching morality and promoting moral sensitivity in nursing students should be further emphasized in Korea (33). Park et al. reported higher levels of moral sensitivity among the senior students in their study compared to the freshmen and asserted that moral education in nursing programs should target the promotion of moral sensitivity and reasoning (21). Comrie studied moral sensitivity in both undergraduate nursing students and nursing graduates and noted the importance of emphasizing moral concepts, especially moral sensitivity, over the course of education (19). Karimi also demonstrated that nursing students in Iran have moderate levels of moral sensitivity and argued that this moral concept plays a major role in providing ethical nursing care (32). Considering that moral sensitivity is a fundamental issue in nursing care that has not been sufficiently investigated, the present study was performed to determine the level of moral sensitivity and its dimensions in Iranian nursing students in 2015.

## Method

The present descriptive study was conducted between April and June 2015 in Qazvin, a city in northern Iran, about 150 km west of the capital (Tehran). Study subjects consisted of eligible undergraduate nursing students from Qazvin University of Medical Sciences selected through census sampling. The census method was chosen because the goal was to determine the level of moral sensitivity in all the undergraduate nursing students of Qazvin University. For this purpose, a list of nursing students was obtained from the Deputy of Education, and the questionnaires were distributed among the students on the list. Study inclusion criteria consisted of being an undergraduate student and having passed at least one academic term. Incomplete or inconsistent information on the questionnaires resulted in exclusion from the study. Of the total of 205 questionnaires distributed among the students, 175 were properly completed and were thus collected for further analysis.

Participants' data were collected using a demographic questionnaire containing items on age, gender, marital status, current academic term, nursing work experience, interest in the nursing profession and previous attendance in ethics classes. Current academic term referred to the semester in which the students were studying at the time of the research by self-report, and work history was concurrent employment in clinical settings. Participants’ moral sensitivity was measured using Lutzen's Moral Sensitivity Questionnaire (MSQ), translated by Abbaszadeh. This questionnaire was developed by Kim Lutzen in Sweden and later modified by Comrie. It contains 25 items on moral sensitivity and is scored based on a Likert scale from 0 (totally agree) to 4 (totally disagree) with a minimum total score of zero and a maximum of 100. A total score between 0 and 50 signifies a low level of moral sensitivity, while scores of 50 to 75 indicate a moderate, and 75 to 100 a high level of moral sensitivity. The validity of the questionnaire was measured using the content validity method through implementing the modifications proposed by ten faculty members of Qazvin University of Medical Sciences who reviewed the items. The reliability of the questionnaire was confirmed using the internal consistency method and by measuring the Cronbach's alpha value (α = 0.82).

The research team began collecting the data after obtaining the necessary permissions and approvals from the Ethics Committee of Shahid Beheshti University of Medical Sciences (No. 6440, dated 11/29/2015). The participating students were briefed on the study objectives and methods of completing the questionnaire before receiving their copy. They were also ensured of the confidentiality of the data and the voluntary nature of participation in the study. All the participants read and signed informed consent forms. The collected data were analyzed in SPSS 16, and the normal distribution of the data was verified using the Shapiro-Wilk test. Descriptive statistics including the mean, percentage and standard deviation were used to describe the data. Moral sensitivity was analyzed through descriptive tests such as the mean, percentage and standard deviation. Pearson’s Correlation Coefficient was used to determine the relationship between age and academic term, and the relationship between moral sensitivity and the other variables was measured by the chi-square test. The level of statistical significance was set at *P* < 0.05.

## Results

The mean age of the participating students was 21.9 with a standard deviation of 2.82. A total of 60% of the participants were female, and the majority were single (90.3%) and had no previous nursing experience (78.9%). Only 6.3% of the students had taken ethics courses before, and the majority (57.7%) had chosen nursing out of interest (Table 1). The mean score of moral sensitivity was 66.1, with a standard deviation of 8.1. The majority of the students (88%) possessed moderate levels of moral sensitivity (Table 2). Of the demographic variables examined, only age was found to have a significant positive correlation with the score of moral sensitivity (r = 0.2 and *P* = 0.01). 

As shown in Table 3, of all the dimensions of moral sensitivity, "expressing benevolence" had the highest mean score (16.9 ± 4.04), while "structuring moral meaning" had the lowest (5.2 ± 1.45); moreover, "relational orientation" was the only dimension that had a significant positive correlation with age (r = 0.2 and *P*<0.01).

**Table 1:** The demographic variables of participants

Variable		Number	Percentage	Variable		Number	Percentage
Gender	Male	70	40	**Previous Attendance in Ethics Classes**	Yes	11	6.3
	Female	105	40		No	164	93.7
Marital Status	Single	158	60	**Academic Term**	Second	32	18.3
	Married	17	9.7		Third	25	14.3
Interest in Nursing	Yes	101	57.5		Fourth	31	17.7
	No	74	42.3		Fifth	22	12.6
Nursing Work Experience	Yes	37	21.1		Sixth	27	15.4
	No	138	78.9		Seventh	10	5.7
					Eighth	28	16

**Table 2:** The absolute and relative frequency and the mean score of moral sensitivity in the study participants

Variable	Level of Moral Sensitivity	Number	Percentage	Mean Overall Score	Standard Deviation
Moral Sensitivity	Low	4	2.3	66.1	8.1
	Moderate	154	88		
	High	17	9.7		

**Table 3:** The mean overall score of the dimensions of moral sensitivity in the study participants

Dimension	Mean	Standard Deviation
Experiencing moral conflict	8.26	1.91
Following the rules	12.5	2.01
Relational orientation	15.38	2.62
Expressing benevolence	16.9	4.04
Modifying autonomy	7.96	1.62
Structuring moral meaning	5.11	1.45

## Discussion

The present study was conducted to determine the level of moral sensitivity and its dimensions in nursing students. The nursing students in this study showed a moderate level of moral sensitivity, which is consistent with the results obtained in previous researches (19, 32-34). The levels of moral sensitivity among nursing students were found to be moderate by Comrie and Abbaszadeh et al., and low to moderate by Ahn and Yeom. These findings suggest that nursing students are relatively familiar with the ethical concepts of patient care, but due to certain barriers, they lack the required sensitivity and suffer from confusion and indifference in relation to moral issues. Therefore, nursing students require further training on ethical concepts in order to be able to apply ethical principles to patient care in their future career. 

Of all the dimensions of moral sensitivity, "expressing benevolence" received the highest, and "structuring moral meaning" the lowest scores. In the MSQ, "expressing benevolence" refers to such concepts as honesty, trust between nurses and patients, considering the patients’ reactions to care, and the patients’ insight and knowledge about their disease. As consistent with the present findings, "expressing benevolence" also received the highest score in a study conducted by Abdou et al. (18) In the study by Comrie, "following the rules" received the highest and "experiencing moral conflict" the lowest scores (19). Nursing students appear to have a greater sensitivity toward "expressing benevolence" because they are still in apprenticeship and therefore pay more attention to theoretical issues and their application in clinical settings. "Structuring moral meaning" is a dimension of moral sensitivity related to issues about which decisions are made without the patient’s participation. This dimension received the lowest score in the present study, which suggests that students still have problems in this area and do not have the right attitude toward the issue. In other words, they do not consider it necessary to involve patients in their medical care decisions. The fact that patients have no defined role in making decisions about their medical care and that the majority of decisions are made by the healthcare personnel including doctors and nurses, may be attributed to the paternalistic view still is dominating the Iranian healthcare system (35, 36).

Of the demographic variables examined, only age had a significant positive correlation with the score of moral sensitivity, as the older students received higher scores. Age was also positively and significantly correlated with "relational orientation", since the older students received higher scores in this area, which includes issues such as the patient-nurse relationship and the patients’ involvement in care. Aging and the subsequent mental maturity of the students, on the one hand, and the greater presence in clinical settings after having passed more courses and moved up the academic ladder, on the other, add to the students’ clinical experience. The outcome is a heightened awareness of the effect of ethics on the quality of nursing care and the best means of building a relationship with the patients, thus increasing nursing students’ level of moral sensitivity (9, 37).

The present study was conducted only on nursing students in Qazvin University of Medical Sciences, and the data were collected through self-report. Therefore, similar studies are needed to be conducted in other nursing schools to establish the generalizability of the results. 

## Conclusion

The moderate level of moral sensitivity in nursing students is indicative of their knowledge about and attention to moral principles in the profession. However, this level of moral sensitivity is not sufficient for providing satisfactory patient care, and promotion of moral sensitivity should therefore be a priority for the nursing system. Holding regular courses on the principles of nursing ethics during the students’ education can certainly enhance their moral sensitivity. Moreover, identification of other factors that can affect the level and dimensions of moral sensitivity is clearly essential. 
